# The Impact of Smoking on Subgingival Plaque and the Development of Periodontitis: A Literature Review

**DOI:** 10.3389/froh.2021.751099

**Published:** 2021-10-27

**Authors:** Jiaxin Zhang, Jialu Yu, Jinge Dou, Pingyue Hu, Qiang Guo

**Affiliations:** ^1^West China School of Stomatology, Sichuan University, Chengdu, China; ^2^State Key Laboratory of Oral Diseases, National Clinical Research Center for Oral Diseases, West China Hospital of Stomatology, Sichuan University, Chengdu, China

**Keywords:** smoking, periodontitis, periodontal microecology, subgingival plaque, subgingival microbiota, periodontopathogen, virulence factor

## Abstract

Smoking seriously affects oral health and causes a variety of oral diseases. Numerous clinical data show that smoking significantly increases the risk of periodontitis, and the duration and amount of smoking are positively correlated with the severity of periodontitis. In fact, smoking creates an environment conducive to the colonization of periodontopathogens, which affects the process of periodontitis. Since subgingival plaque which harbors periodontopathogens is the initiation factor of periodontitis, it is critical to study the impact of smoking on subgingival microbiota for understanding the relationship between smoking and periodontitis. Continuous advances have been made on the understanding of effects of smoking on subgingival plaque and the development of periodontitis. Smoking is observed to enhance the pathogenicity of periodontopathogens, especially the red complex microorganisms, via promoting their colonization and infection, and regulating the expression and function of multiple virulence factors. Furthermore, smoking has a negative impact on periodontal microecological homeostasis, which is reflected in the decrease of commensal bacteria and the increase of periodontopathogens, as well as the changes in the interaction between periodontopathogens and their commensal microbes in subgingival biofilm, thus influencing the pathogenicity of the subgingival plaque. In summary, the mechanism of smoking on subgingival plaque microorganisms represented by the red complex and its effect on the periodontal microecology still need to be further explored. The relevant research results are of great significance for guiding the periodontal clinical treatment of smoking population. This review summarizes the effects and relevant mechanisms of smoking on subgingival plaque and the development of periodontitis.

## Introduction

Periodontitis is an inflammatory and destructive disease involving periodontal supporting tissue, including gingiva, periodontal ligament, alveolar bone and cementum [1]. So far, periodontitis is known as the sixth most common human disease because of its high prevalence and great influence [2]. The WHO report shows that the periodontal condition is poor worldwide, with almost 10% of the global population affected severe periodontitis [3]. The results of National Health and Nutrition Examination Surveys (2009–2014) show that 42.2% American adults aged 30–79 suffer from periodontitis, of which 7.8% suffer from severe periodontitis [4]. In 2019, there were 1.1 billion cases of severe periodontitis worldwide. The age-standardized prevalence rate in 2019 was 13109/100000. From 1990 to 2019, the global age-standardized prevalence rate increased by 8.44% [5]. Periodontitis is also related to systemic diseases, such as cardiovascular disease, rheumatoid arthritis, respiratory disease, resulting in more serious effects on general health [2, 6–8]. Therefore, at present, much attention has been paid to the research on pathogenesis, prevention and treatment of periodontitis.

Periodontitis is a multifactorial disease caused by the imbalance among microbes, host and environment. Subgingival plaque and its products are the initiating factors of periodontitis, both of which are closely related to the occurrence and development of periodontitis [9]. When the invasion of microbes and the defense function of the host maintain the dynamic equilibrium of the periodontal microecology, the pathogenic effect of a small number of periodontopathogens can be defended by the immune function of the host [1]. However, some cytokines, prostaglandins and matrix metalloproteinases produced during the disorder of host inflammatory response can mediate the destruction of periodontal tissues and eventually lead to periodontitis [10–12]. There are also some general and local pathogenic factors of periodontitis, such as genetic factors, systemic diseases, dental calculus, anatomical abnormalities, malocclusion, and poor restorations [13, 14]. At the same time, some specific systemic diseases are found to contribute to different types of periodontitis such as aggressive periodontitis [15].

Smoking is a risk factor for a variety of systemic diseases, including lung disease, cardiovascular disease and so on [16–18]. Some studies have shown that smoking can lead to oral dysbacteriosis [19], so it is related to the initiation and development of oral diseases driven by oral microflora, such as periodontitis [20], dental caries [21], periapical periodontitis [22], peri-implantitis [23]. In addition, a link between the occurrence and progression of oral cancer and smoking is also observed [24, 25]. Among the oral diseases related to smoking, periodontitis has been demonstrated to have a strong association with smoking [20, 26, 27]. In fact, smoking is the second highest risk factor for periodontitis [28]. Although the mechanisms that smoking involves in the progress of periodontitis are not fully understood, based on the special relationship between subgingival plaque and periodontitis, one critical mechanism is that smoking influences the balance of subgingival plaque microbiota and periodontal microecology [29, 30]. Here, this review will summarize the effects and relevant mechanisms of smoking on subgingival plaque and the development of periodontitis.

## Clinical Correlations Between Smoking and Periodontitis

A number of clinical studies have shown that smoking is closely related to the occurrence, development and severity of periodontitis. By analyzing the general situation of periodontitis in smokers and non-smokers, it was found that smoking habits increased the risk of periodontitis by 90% [31]. At the same time, attribution analysis showed that 74.8% of periodontitis cases in the United States could be attributed to smoking [26]. Studies have found that current smokers are more likely to suffer from periodontitis, while severe periodontitis is more common among current smokers [32].

The gingival condition and probing situation reflect the periodontal status and are often used in the diagnosis of periodontitis. Through oral examination, it is found that there are some significant differences in the periodontal clinical indexes, including plaque index (PI), probing depth (PD) and clinical attachment loss (CAL), between smokers and non-smokers. Some studies have shown that the PI, PD and CAL values in smokers are higher than those in non-smokers [33]. At the same time, the values of PD and CAL are correlated with the concentrations of metabolites of nicotine [33, 34]. On the other hand, the periodontal examination of smokers showed that 93.6% of smokers had one or more CAL > 3 mm [32]. While severe attachment loss, that is, CAL > 7 mm, is also the most popular and appeared in current smokers (27.0%) [32]. At the same time, severe probing depth PD > 7 mm is also the most common among current smokers [32].

The beneficial effects of smoking cessation on periodontal health also suggest the adverse effects of smoking on the prevention and treatment of periodontitis. As far as we know, smoking cessation is an important preventive measure for periodontitis and a necessary adjunct in the treatment of periodontitis. First of all, smoking cessation reduces the incidence of periodontitis. One report showed no significant difference in the prevalence of periodontitis in those who quitted smoking compared to non-smokers, and both were lower than smokers [35]. Smoking cessation can also influence the progressive course of periodontitis. After the assessment of the periodontal status of smokers, non-smokers and quitters, it was found that the periodontal status of quitters was between the other two [36, 37]. Some studies pointed out that certain harmful effects of smoking on periodontium were reversible in the case of smoking cessation [38]. Fiorini et al. [39] found through a systematic review that obvious reversal of periodontitis risk in smokers could be achieved within 10 years after quitting.

In addition to reducing the incidence and risk of progression of periodontitis, smoking cessation is very beneficial to periodontal treatment, especially for non-surgical treatment [40]. In the short term, quitters and non-smokers showed similar responses to periodontal treatment [41]. The degree of changes in PD, CAL and alveolar bone level was similar in quitters and non-smokers under long-term observation and both were lower than smokers [42]. Even after non-surgical treatment, smoking cessation led to reductions in PD and CAL compared to smokers [42–44]. Smoking cessation is also effective in reducing the risk of tooth loss, as Maria Luisa Silveira Souto et al. [45] found through a systematic review and Meta-analysis that the risk of tooth loss in quitters was reduced compared to smokers (RR = 2.60), and was comparable to that of non-smokers (RR = 1.15).

## Impact of Smoking on Pathogenicity of Periodontopathogens

The main microorganisms in subgingival plaque can be divided into six different periodontal microbial complexes according to their colonization, distribution in microflora and the relationship with periodontal status [46]. Among them, the red microbial complex is most closely related to periodontitis, including *Porphyromonas gingivalis, Tannerella forsythia* and *Treponema denticola* [47]. *P. gingivalis* is believed to play a key and pioneering role in the progression of periodontitis [21]. One of the main mechanisms of smoking affecting periodontitis is that cigarette smoke and tobacco derivatives affect the pathogenicity of periodontopathogens, especially *P. gingivalis*.

### Growth and Colonization of Periodontopathogens

Multiple studies have revealed that smokers are more susceptible than non-smokers to persistent *P. gingivalis* infection [48–52]. As a common toxic ingredient in cigarettes, nicotine can inhibit the growth of *P. gingivalis* in a short time, but *P. gingivalis* can develop resistance to nicotine in a very short time, and then increase the growth under such a condition [53]. Smoking is thought to create a more anoxic environment, which may favor the growth and colonization of the obligate anaerobes such as *P. gingivalis* [54, 55]. In addition, under the stimulation of tobacco derivatives such as nicotine and cotinine, the expression of oxidative stress-related proteins in *P. gingivalis* is up-regulated [56], so that *P. gingivalis* can survive under oxygen exposure, suggesting the strong adaptability and survival ability of the periodontopathogen to different environmental stress. Biofilm formation of *P. gingivalis* was also observed to be augmented under the stimulation of cigarette smoke extract (CSE), with a significant increase in biomass, substratum coverage, and maximum and mean thickness apparent [52]. It is notable that CSE-treated *P. gingivalis* biofilms exhibited a lower pro-inflammatory capacity (TNF-α, IL-6) than control biofilms, which may explain the increased persistence of this pathogen in smokers [57].

The influence of tobacco derivatives on epithelial colonization by periodontopathogens have been reported. Nicotine and cotinine can promote the colonization of the periodontopathogen *Aggregatibacter actinomycetemcomitans* to epithelial cells at the concentration of 1 mg/ml [58]. However, no positive effect of nicotine on epithelial colonization of *P. gingivalis* was observed, and the invasiveness of *P. gingivalis* increases significantly at a concentration of cotinine (100 μg/ml) [59]. Take into consideration that concentration of nicotine and cotinine found in oral cavity of smoking patients is very low [60], whether oral epithelial cells of smokers are more likely to be colonized by periodontopathogens needs to be further studied.

### Expression and Function of Virulence Factors of Periodontopathogens

**Figure 1:**
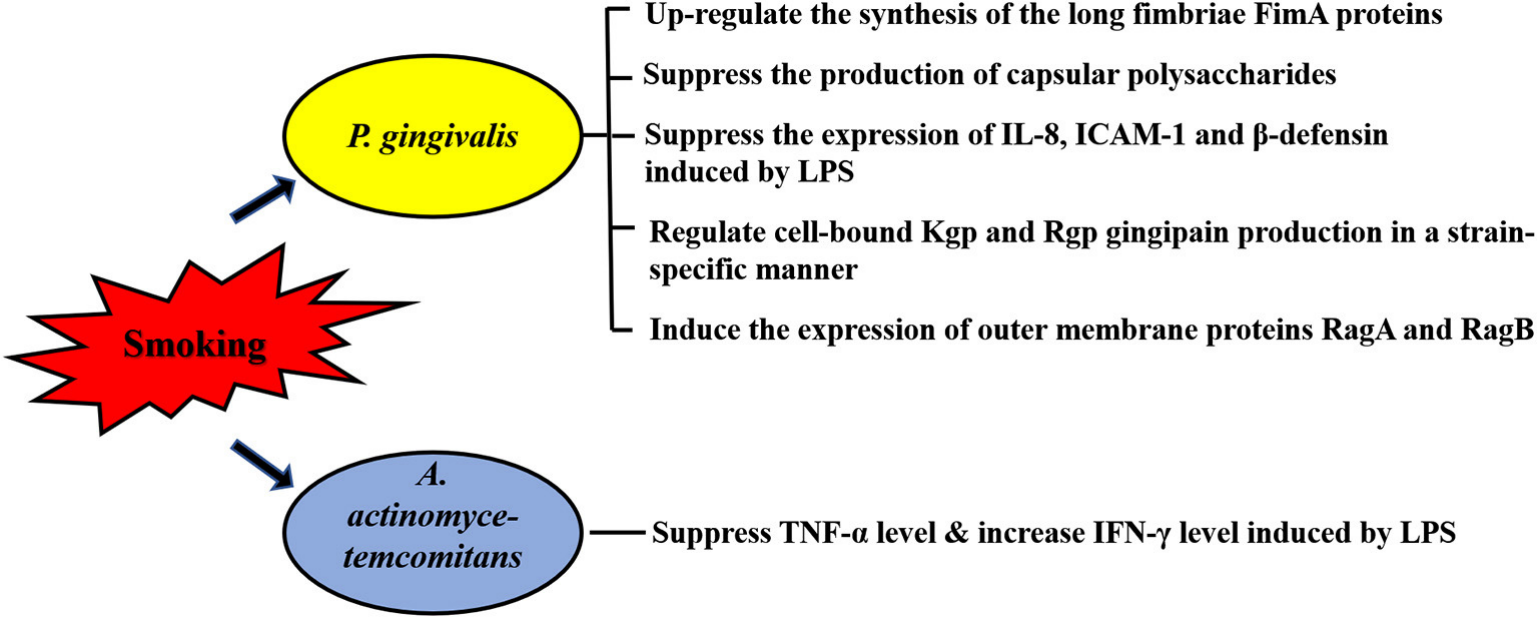
Effects of smoking on virulence factors of periodontopathogens. Expression or function of multiple virulence factors of *P. gingivalis* are affected by smoking, including the long fimbriae FimA, capsular polysaccharides, outer membrane proteins RagA and RagB, Kgp and Rgp gingipain and lipopolysaccharide (LPS). The immune response caused by LPS of *A. actinomycetemcomitans* is also influenced by smoking. Intercellular adhesion molecule-1, ICAM-1.

Periodontopathogens can produce various virulence factors, such as extracellular proteases, lipopolysaccharide (LPS) and metabolites, which contact or enter the periodontium to directly destroy cells, or cause local immune and inflammatory response in periodontal tissue to cause tissue damage indirectly. Thus, the role of virulence factors of periodontopathogens is particularly important in the progress of periodontitis, especially for *P. gingivalis*, which possesses many virulence factors. Using microarrays representative of the *P. gingivalis* genome, it is revealed that CSE-exposure resulted in differential regulation of 6.8% of *P. gingivalis* genes, including detoxification and oxidative stress-related genes, DNA repair genes and multiple genes related to *P. gingivalis* virulence [61]. More studies demonstrated the multiple regulatory effects of smoking on expression or function of virulence factors of *P. gingivalis*, including the long fimbriae FimA [52, 61], capsular polysaccharides [52], outer membrane proteins RagA and RagB [61], Kgp and Rgp gingipain [62] and LPS [63] (Figure 1).

In addition of alterations in gene express profile, a reduced proinflammatory potential is observed in the response of *P. gingivalis* to CSE. The long fimbriae FimA is one of the main factors responsible for the colonization of the oral cavity by *P. gingivalis* [64]. Bagaitkar et al. found CSE exposure could up-regulate the synthesis of FimA proteins which induce TLR2 hyposensitivity, thus reducing the host response to *P. gingivalis* [52]. Given that the production of capsular polysaccharides and the expression of IL-8, intercellular adhesion molecule (ICAM)-1 and β-defensin induced by *P. gingivalis* LPS are also suppressed by CSE, it is suggested that smoking represents an environmental stress which may promote *P. gingivalis* colonization and infection via reducing the pro-inflammatory cytokine burden inducing by the virulence factors of the pathogen [52, 62]. CSE also affects the immune response caused by LPS of *A. actinomycetemcomitans*. An *in vivo* study reported that both 10 μg and 200 μg nicotine significantly reduced TNF-α levels induced by *A. actinomycetemcomitans* LPS, but only 200 μg nicotine-treatment resulted in a higher level of IFN-γ induced by *A. actinomycetemcomitans* LPS [65] (Figure 1).

## Impact of Smoking on Periodontal Microecology

Periodontal microecosystem consists of periodontal microbiota, periodontium and various environmental factors including nutrients, temperature, oxygen, pH, etc., as well as interactions between periodontal microbiota and between periodontal microbiota and their hosts. The balance of periodontal microecology can be disturbed by multiple factors from host, microbiota or environment, in which smoking is identified as an important risk factor for homeostasis of periodontal microecology.

### A More Anaerobic Environment

Smoking may create a more anaerobic environment in the oral cavity and thus affect the periodontal microecology. Smoking causes gingival vasoconstriction in patients with periodontitis and the pocket oxygen tension of smokers is lower than that of non-smokers [66, 67]. Wu J et al. found that the expression of signal pathways related to aerobic metabolism (the tricarboxylic acid cycle and oxidative phosphorylation of tricarboxylic acid) was reduced in the oral bacteria of smokers, while the expression of non-oxygen metabolic pathways (glycolysis, fructose, galactose and sucrose metabolism and photosynthesis) was enhanced compared to non-smokers [19]. Moreover, decreased local oxygen tension caused by smoking is likely to promote the growth of anaerobic periodontal pathogens such as *Fusobacterium, Treponema, P. gingivalis*, influencing periodontal microbiota [29, 68–71].

### Abundance and Diversity of Subgingival Microbiota

An anaerobic environment caused by smoking has a pronounced impact on the subgingival microbiota, especially on the ratio of anaerobes to aerobes. Compared with non-smokers, the abundance of anaerobic bacteria in subgingival plaque samples of smokers was significant higher, while the abundance of aerobic bacteria was lower [72]. Many studies also showed that smokers were infected with a higher proportion of anaerobic periodontopathogens such as *Fusobacterium, Treponema, P. gingivalis, Tannerella forsythia* and other periodontal pathogenic bacteria [29, 68–71]. In fact, it is known that subgingival microbial profile is compositionally different in current and never-smokers, with significant differences in the abundance and diversity of subgingival microbiota, although the research results vary from study to study (Table 1). For example, in 2011, Kumar et al. [68] showed that the subgingival biofilm of smokers exhibited a greater diversity in the early stage of subgingival plaque formation (within 7 days) compared with non-smokers, although a decrease in diversity over 7 days was observed. However, in 2015, Camelo-Castillo et al. reported that the bacterial diversity of smoking periodontal patients was higher than that of non-smoking healthy controls but lower than that of non-smoking periodontal patients, after comparing the composition of their subgingival microbiota [55]. In the study of Wu et al., 16S rRNA gene sequencing revealed a lower relative abundance of the phylum *Proteobacteria* in current smokers (4.6%), compared with never smokers (11.7%), which was also demonstrated at class, genus and operational taxonomic unit (OTU) levels [19]. Moreover, the genera *Capnocytophaga, Peptostreptococcus* and *Leptotrichia* were also depleted, while *Atopobium* and *Streptococcus* were enriched in smokers [19].

**Table 1:** Clinical studies on the effects of smoking on abundance and diversity of subgingival microbiota (published between 2006 and 2021).

**Year & Reference**	**Periodontal condition**	**Laboratory techniques**	**Microbes targeted**	**Main results**	
				**Smokers vs. non-smokers**	**Smokers vs. ex-smokers**
2009, [73]	CP	t-RFLP	Community	NA	Microbial profiles differed significantly between smokers and quitters at 6 and 12 months following smoking cessation.
2010, [74]	CP	PCR	Community	NA	Following NPT and smoking cessation, the subgingival microbiome was recolonized by a greater number of health-associated species and there were significantly lower prevalence and abundance of putative periodontal pathogens.
2010, [29]	CP	16S sequencing	Community	Greater levels of the genera *Parvimonas, Campylobacter, Treponema, Bacteroides* and *Fusobacterium in smokers*, and higher levels of *Streptococcus, Veillonella*, and *Neisseria* in non-smokers	NA
2011, [68]	Healthy	16S sequencing	Community	A highly diverse, relatively unstable initial colonization of subgingival biofilms in smokers, with a lower niche saturation than that in non-smokers.	NA
2015, [72]	Healthy	16S sequencing	Community	A highly diverse, pathogen-rich, commensal-poor, anaerobic microbiome in smokers.	NA
2015, [55]	Healthy, CP	16S sequencing	Community	The microbial community of smoking periodontal patients was less diverse that of non-smoking periodontal patients, but more diverse than that of non-smoking healthy controls.	NA
2016, [19]	Healthy, periodontitis	16S sequencing	Community	Reduced abundance of *Proteobacteria* and enriched *Firmicutes* and *Actinobacteria* in smokers.	NA
2016, [70]	Before and 3, 6 months after NPT	PCR	*Aa, Pg, Tf, Ca, Cd, Cg, Ct*	Only *Aa* was statistically higher at baseline (pretherapy) in smokers compared to non-smokers.	NA
2017, [69]	Healthy, CP	PCR	25 bacterial species	Smoking results in the depletion of beneficial bacteria and the increase in periodontal pathogenic bacteria.	NA
2019, [34]	CP	PCR	*Td, Pg*	The frequency of *Pg* was found to be similar in the two groups, and *Td* was more frequently detected in the smoker but the difference was not significant.	NA

*CP, chronic periodontitis; t-RFLP, terminal restriction fragment length polymorphism; PCR, polymerase chain reaction; NPT, non-surgical periodontal therapy; Aa, Aggregatibacter actinomycetemcomitans; Pg, Porphyromonas gingivalis; Tf, Tannerella forsythia; Ca, Candida albicans; Cd, Candida dubliniensis; Cg, Candida glabrata; Ct, Candida tropicalis; Td, Treponema denticola*.

In the periodontal microecosystem, commensal bacteria exert antagonistic effects on periodontopathogens by secreting antimicrobial substances, competing with pathogens for mucosal surface binding sites, and adjusting environmental pH, so as to maintain the stability of periodontal microecology [75, 76]. However, smoking is found to reduce the abundance of commensal microbes in subgingival plaque and thus change the composition of subgingival microbiota. The numbers of health-related *Veillonella, Neisseria* and some kinds of *Streptococcus* such as *Streptococcus sanguis* decrease significantly in subgingival biofilm of smokers [29].

Besides, alterations in subgingival microbiota could result from smoking cessation (Table 1). The subgingival plaques of smokers and people who had quit smoking for 3, 6, 12 months were compared and significant differences in microbial distribution and species diversity were found between them [73]. A more in-depth study by Delima et al. found that after 12 months of smoking cessation, the prevalence of *Porphyromonas endodontalis* and *Dialister pneumosintes* and the levels of *Parvimonas micra, Filifactor alocis* and *Treponema denticola* were all reduced in subgingival plaque, but *Veillonella parvula* levels were increased [74]. However, studies addressing the mechanism of smoking cessation on subgingival biofilm are scarce and the exact mechanism remains unclear.

### Interspecies Interaction in Subgingival Plaque

**Figure 2:**
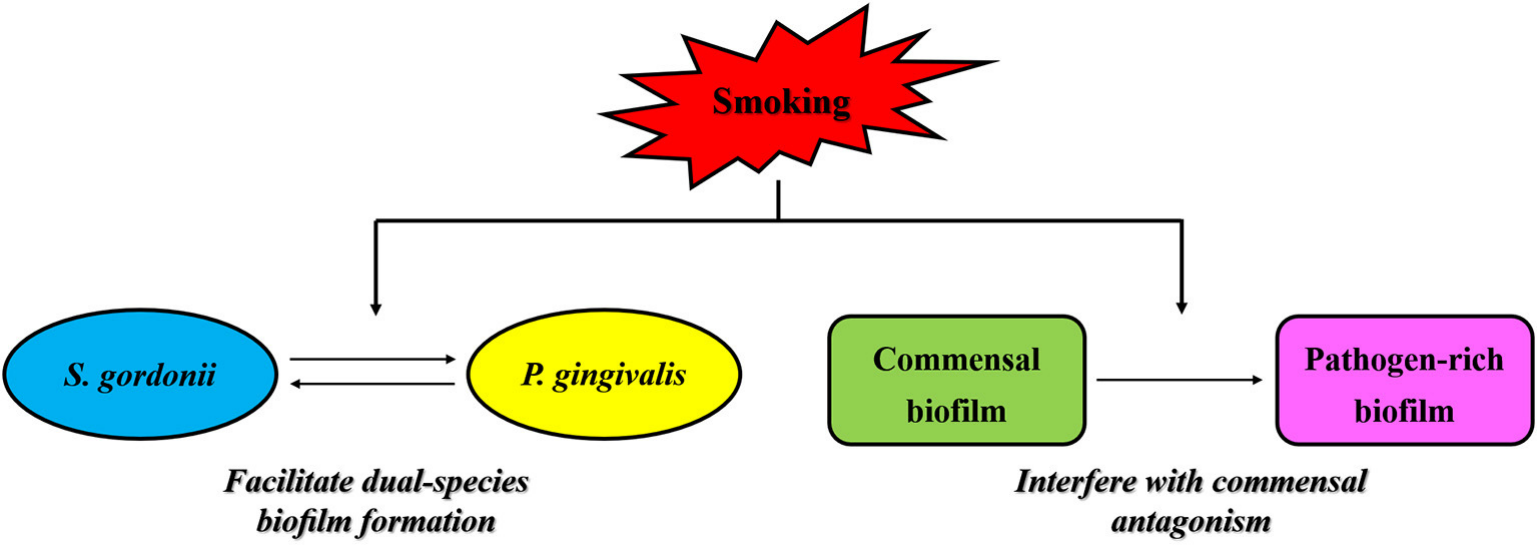
Effects of smoking on interspecies interaction in subgingival plaque. Smoking is reported to facilitate *P. gingivalis*-*S. gordonii* dual-species biofilm formation via enhancing the binding of *P. gingivalis* FimA to glyceraldehyde-3 phosphate dehydrogenase, the cognate FimA ligand on *S. gordonii*, in a dose-dependent manner. Moreover, smoking is observed to interfere with the antagonistic effects of commensal microbes on periodontal pathogens via a mechanism in which smoke exposure could induce significant transcriptional shifts in commensal biofilms and trigger a florid pro-inflammatory response leading to early commensal death, thus contributing to the formation of pathogen-rich subgingival biofilms and the subsequent development of periodontitis in smokers.

In the process of plaque maturation, streptococci like *Streptococcus gordonii* that colonize in the early stage provide binding sites for later colonized bacteria such as *P. gingivalis*, allowing them to attach and form mature biofilms [77]. Huang et al. [78] reported that the stimulation of nicotine enhanced cell growth, biofilm formation and cell aggregation of *S. gordonii*, as well as up-regulating the expression of 11 genes that encode binding proteins or regulators. These effects of nicotine may enhance the binding and colonization of *P. gingivalis* in oral cavity, and further promote the development of periodontitis in cigarette smokers. More interestingly, CSE is reported to facilitate *P. gingivalis*-*S. gordonii* dual-species biofilm formation in a FimA-dependent manner [57] (Figure 2). It is observed that compared to control biofilms, CSE treatment significantly enhanced the binding of *P. gingivalis* FimA to glyceraldehyde-3 phosphate dehydrogenase (GAPDH), the cognate FimA ligand on *S. gordonii*, in a dose-dependent manner [57]. CSE exposure also results in an approximately two-fold increase in the total number of *P. gingivalis*-*S. gordonii* microcolonies and a three-fold increase in the microcolony height as compared with the control biofilms without exposure to CSE [57].

In addition to enhancing the interspecies interaction between *P. gingivalis* and *S. gordonii*, smoking is also observed to interfere with the antagonistic effects of commensal microbes on periodontal pathogens, thus contributing to the formation of pathogen-rich subgingival biofilms and the subsequent development of periodontitis in smokers. It is found by Kumar et al. that oral biofilms in clinically healthy smokers are pathogen-rich and commensal-poor, and this pathogen enrichment occurs within 24 h of biofilm formation [68]. To identify the potential mechanism by which smoking creates this altered community structure, they conducted a further study showing that smoke exposure induced significant transcriptional shifts in commensal biofilms, with suppressed essential metabolic functions and increased expression of virulence genes such as LPS, flagella and capsule synthesis, and then triggered a florid pro-inflammatory response, leading to early commensal death, which might preclude niche saturation by commensal microbes and thus, pathogen-rich biofilms in smokers could be formed in the absence of commensal antagonism [79] (Figure 2).

## Summary and Outlook

Smoking is a common etiology and risk factor for many diseases throughout the body. For periodontitis, smoking is a critical risk factor, even as important as bacteria for patients with severe periodontitis. Therefore, it is essential to study the mechanisms by which smoking affects the occurrence and development of periodontitis. A brief overview of the effect of smoking on the course of periodontitis can be obtained from the current literature. Firstly, smoking increases the susceptibility of patients to infection of periodontopathogens. Secondly, smoking accelerates the progression of periodontitis via accelerating the destruction of periodontal supporting tissues, which increases the severity of periodontitis. Smoking also hinders the treatment of periodontitis patients and facilitates its recurrence. Overall, the effects of smoking on subgingival bacteria-host interactions are key to these changes, including the effects of smoking on host cells, blood vessels, etc. and immunoinflammatory processes, as well as the effects of smoking on periodontal microecology and periodontal microbial interactions. Although a large amount of literature has been reported, the relevant mechanisms are still less clear, especially with regard to the mechanisms of action of smoking on subgingival microbiota. Even different literature has shown different results in studies on the detection rate of periodontal pathogenic bacteria in smokers and non-smokers and the impact of smoking on the abundance and diversity of subgingival microbiota. Moreover, the differences in the results of studies on the effect of smoking on periodontopathogens caused by different *in vivo* and *in vitro* environments are still a challenge to be overcome. In-depth studies on the mechanisms by which smoking affects subgingival plaque and aggravates periodontitis will help to deepen our understanding of the pathogenesis and influence factors of periodontitis, and also help to continue to explore more effective prevention and treatment measures for periodontitis in patients who smoke.

## Author Contributions

JZ and QG conceptualized the review. JZ, JY, and JD drafted the manuscript and QG edited the manuscript, with PH providing critical revisions. All authors contributed significantly, read, and approved the final manuscript.

## Funding

This work was supported by grants from the Science and Technology Department of Sichuan Province (Grant No. 2021YJ0133) and the Undergraduate Innovation and Training Program of Sichuan University (Grant Nos. C2020112072 and C2021118018).

## Conflict of Interest

The authors declare that the research was conducted in the absence of any commercial or financial relationships that could be construed as a potential conflict of interest.

## Publisher's Note

All claims expressed in this article are solely those of the authors and do not necessarily represent those of their affiliated organizations, or those of the publisher, the editors and the reviewers. Any product that may be evaluated in this article, or claim that may be made by its manufacturer, is not guaranteed or endorsed by the publisher.
